# Long-term, health-enhancing physical activity is associated with reduction of pain but not pain sensitivity or improved exercise-induced hypoalgesia in persons with rheumatoid arthritis

**DOI:** 10.1186/s13075-018-1758-x

**Published:** 2018-11-26

**Authors:** Monika Löfgren, Christina H. Opava, Ingrid Demmelmaier, Cecilia Fridén, Ingrid E. Lundberg, Birgitta Nordgren, Eva Kosek

**Affiliations:** 1 Department of Clinical Sciences, Danderyd Hospital, Karolinska Institutet, SE-182 88 Stockholm, Sweden; 2 Department of Rehabilitation Medicine, Danderyd Hospital, Building 60, SE-182 88 Stockholm, Sweden; 3 Division of Physiotherapy, Department of Neurobiology, Care Sciences and Society, Karolinska Institutet, Alfred Nobels Allé 23, SE-141 83 Huddinge, Sweden; 4 Rheumatology Clinic, Karolinska University Hospital, SE-171 76 Stockholm, Sweden; 5 Division of Rheumatology, Department of Medicine, Solna, Karolinska Institutet, D2:01, SE-171 76 Stockholm, Sweden; 6 Functional Area Occupational Therapy and Physiotherapy, Allied Health Professionals Function, Karolinska University Hospital, D4:51, SE-171 76 Stockholm, Sweden; 7 Department of Clinical Neuroscience, Karolinska Institutet, Nobels väg 9, SE-171 77 Stockholm, Sweden; 8 Department of Neuroradiology, Karolinska University Hospital, SE-171 76 Stockholm, Sweden; 9 Stockholm Spine Center, Löwenströms väg 1, SE-194 89 Upplands Väsby, Sweden

**Keywords:** Arthritis, Exercise-induced hypoalgesia, Pain measurement, Pain threshold, Long-term follow-up

## Abstract

**Background:**

We aimed to evaluate the 1-year and 2-year outcome of a health-enhancing physical activity (HEPA) support program on global pain, pressure pain sensitivity, and exercise-induced segmental and plurisegmental hypoalgesia (EIH) in persons with rheumatoid arthritis (RA).

**Methods:**

Thirty participants (27 women and 3 men) were recruited from a larger intervention cohort that engaged in strength training and moderate-intensity aerobic activity. Assessments were performed before the HEPA intervention and at 1-year and 2-year follow-ups. Global pain was assessed on a visual analogue scale (0–100). Pressure pain thresholds (PPTs) and suprathreshold pressure pain at rest corresponding to 4/10 (medium pain) (SP4) and 7/10 (strong pain) (SP7) on Borg CR 10 scale were assessed by algometry. In a subsample (*n* = 21), segmental and plurisegmental EIH were assessed during standardized submaximal static contraction (30% of the individual maximum), by algometry, alternately at the contracting right *M. quadriceps* and the resting left *M. deltoideus*.

**Results:**

Global pain decreased from before the intervention to 2-year follow-up (median 11 to median 6, *P* = 0.040). PPTs and SP4 pressure pain at rest did not change from before the intervention to 2-year follow-up, while SP7 decreased from mean 647 kPa to mean 560 kPa (*P* = 0.006). Segmental EIH during static muscle contraction increased from the assessment before the intervention (from mean 1.02 to mean 1.42, *P* = 0.001), as did plurisegmental EIH (from mean 0.87 to mean 1.41, *P* <0.001). There were no statistically significant changes in segmental or plurisegmental EIH from before the intervention to 2-year follow-up.

**Conclusion:**

Participation in a long-term HEPA support program was associated with reduced global pain, whereas pressure pain sensitivity at rest was not reduced and EIH did not change. Thus, our results do not favor the hypothesis that long-term HEPA reduces pain by improving descending pain inhibition in persons with RA.

**Trial registration:**

ISRCTN25539102, ISRCTN registry, date assigned March 4, 2011. The trial was retrospectively registered.

## Background

Physical activity and exercise are central to the treatment of rheumatoid arthritis (RA). Health-enhancing physical activity (HEPA) is recommended to the population at large in order to maintain health and prevent comorbidity, including the risk of cardiovascular disease [1], which is also common in RA and contributes to premature death and poor health in this subgroup of the population.

Recommendations for HEPA include at least 150 min per week of moderate-intensity physical activity and strength training twice weekly [1]. Previous recommendations are also reflected in the recent European League Against Rheumatism (EULAR) recommendations for physical activity in people with inflammatory arthritis and also highlight that such interventions should include behavioral change techniques and that alternative modes of delivery should be considered [2].

Individuals with RA have lower levels of physical activity than the population at large [3] and frequently do not reach recommended HEPA levels [4–6]. In our recent study, 70% of participants with RA reported compliance with HEPA but only 22% had maintained it for at least 6 months [7].

New drugs and updated treatment recommendations have improved control of inflammation among individuals with RA, but many still have chronic pain, disability, and increased risk of cardiovascular disease [8, 9]. HEPA might serve as an important complement to pharmacological treatment with few adverse effects [10]. The promotion of HEPA among individuals with RA offers several benefits, including increased level of physical activity [11], improved perception of health, and greater muscle strength [12]. Pain reduction is often observed following physical activity interventions in RA [13], but the mechanisms behind such effects have not been fully explored.

Several causes of non-inflammatory pain are present; peripheral joint damage induces peripheral sensitization [13] and central sensitization has been documented [14, 15]. A generalized increased pain sensitivity (that is, also outside inflamed joints) has previously been reported in patients with RA [15–17]. Pain sensitivity seems to increase with the duration of RA [14], suggesting progression of central sensitization [15].

Exercise-induced hypoalgesia (EIH) is a top-down pain inhibitory mechanism normally activated during muscle contraction. In healthy individuals, EIH is reflected as an increase in pressure pain thresholds (PPTs) during muscle contraction [18]. We have previously reported increased pain sensitivity to threshold and suprathreshold pressure stimuli at rest but a normal function of EIH during muscle contraction among individuals with RA [16, 17]. Normal EIH was also found among individuals with RA when endogenous pain modulation (the ability of the nervous system to enhance and inhibit the pain experience by different processes) was explored in direct response to submaximal exercise on a bike ergometer [19]. There is currently strong support for the hypothesis that individuals with RA have increased pain sensitivity but a normal function of EIH.

Exercise for RA results in improvements in pain and function [20, 21]. Given that previous studies have reported reduced pain sensitivity and more pronounced EIH [22, 23] among athletes and physically active individuals, it is reasonable to assume that the beneficial effects of long-term HEPA could be mediated by improved top-down pain modulation. We hypothesized that long-term HEPA would reduce pain as well as pain sensitivity by improving the function of endogenous pain modulatory mechanisms, such as EIH. To our knowledge, no previous study has explored the long-term effects of HEPA on pain sensitivity and endogenous pain modulation among individuals with RA.

## Methods

The aim of the present study was to evaluate the 1-year and 2-year outcome of HEPA for global pain, pain sensitivity, and EIH in a subgroup with RA who participated in a HEPA support program [24].

### Participants

**Fig. 1:**
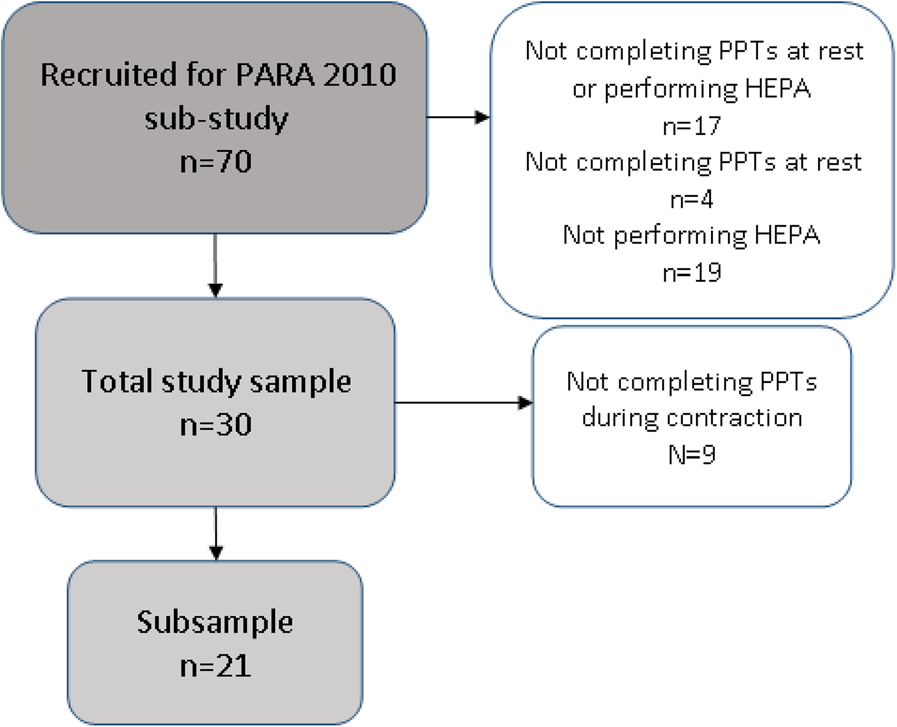
Flowchart of the study sample, the subsample, and dropouts. Abbreviations: *HEPA* health-enhancing physical activity, *PARA* Physical Activity in Rheumatoid Arthritis, *PPT* pressure pain threshold

Thirty participants (90% were women, mean age was 61, and mean disease duration was 12 years) of the 70 originally recruited for investigation with algometry within the 2-year prospective multicenter intervention study of a HEPA support program [17, 24] were eligible for the present follow-up. They represented those who had completed the assessments of PPTs at rest before the intervention and at 1-year and 2-year follow-ups and reached recommended HEPA levels at follow-ups after 1 and 2 years. A subsample (*n* = 21) also completed the assessments of EIH at the same times (Fig. 1 and Table 1). There were no statistically significant differences at the assessments before HEPA in background data or in results of PPTs at rest between the participants in the present study (*n* = 30) and those with incomplete data (*n* = 40).

**Table 1:** Characteristics and self-reported baseline data of study participants

	Total sample *n* = 30	Subsample *n* = 21
Age in years, mean (SD)	61 (10)	60 (11)
Women, n (%)	27 (90)	20 (95)
Disease duration in years, mean (SD)	12 (11)	12 (12)
HRQoL (EQ-5D thermometer) on a scale of 0 to 100, median (25th–75th percentiles)	80 (66–82)	80 (62–80)
Activity limitation (HAQ-DI) on a scale of 0 to 3, median (25th–75th percentiles)	0.38 (0.00–0.88)	0.50 (0.00–0.88)
Current HEPA (IPAQ), responders, n (%)	23 (77)	17 (81)
BMI in kg/m^2^, mean (SD)	25 (3)	25 (3)

Abbreviations: *BMI* body mass index, *EQ-5D* EuroQol-5 dimensions, *HAQ-DI* Stanford Health Assessment Questionnaire Disability Index, *HEPA* health-enhancing physical activity, *HRQoL* health-related quality of life, *IPAQ* International Physical Activity Questionnaire

All participants in the Physical Activity in Rheumatoid Arthritis (PARA) 2010 study were identified through the Swedish Rheumatology Quality Registers and were eligible if 18–75 years old, independent in daily living (Stanford Health Assessment Questionnaire Disability Index, HAQ-DI of not more than 2), interested in participating in organized physical activity, fluent in Swedish, and not currently obtaining maintained HEPA levels for at least 6 months [24].

### HEPA support program

The intervention program included three main components to promote HEPA according to recommendations: (a) at least two weekly strength training sessions (50–60% of one repetition maximum and 3 × 10 repetitions for major muscle groups); (b) physical activity of at least moderate intensity (40–60% of VO_2max_) for at least 30 min during the five remaining days of the week and in order to support HEPA; and (c) support group sessions using weekly goal-setting, planning, and follow-up according to social cognitive theory [25]. Strength (circuit) training was provided at assigned centers. Study participants committed to pay a fee for a 1-year membership, allowing them to drop in whenever they wanted when the center was open. At the start of the 2-year intervention, a physiotherapist instructed each participant about how to perform efficient exercise and was available for consultations once a week during the first year. For the second year, membership could be renewed at the assigned centers or at any other training facility. Pedometers and access to a website for step registration were provided to each participant, and a self-administered walk test to monitor aerobic capacity was taught and recommended [24]. Participants reported weekly frequency of physical activity and circuit training in short texts during the 2-year study period. More details about the program were presented in our previous publication [24].

### Assessments

#### Questionnaires

For descriptive purposes, the following questionnaire data were selected among those collected before start of the HEPA support program of the PARA study [24]:

1. Health-related quality of life as measured by the EuroQol five-dimension (EQ5-D) thermometer assessing perceived health the day in question from “Worst imaginable state of health” (score of 0) to “Best imaginable state of health” (100) [26].
2. Activity limitation as measured by HAQ-DI comprising 20 questions addressing activities of daily living performed within the past week: dressing and grooming, arising, eating, performing personal hygiene, reaching, gripping, walking, and engaging in common daily activities. Each item and a total score may vary between “With no difficulty” (0) and “Unable to perform” (3) [27].
3. Current HEPA as measured by the International Physical Activity Questionnaire (IPAQ) short version, a self-administered questionnaire about physical activity at several intensity levels and across the domains of home, work, transport, and leisure, undertaken over the past 7 days before the assessment [28]. For the present study, IPAQ answers were dichotomized to indicate whether or not participants reached HEPA levels or not (responders/non-responders).

#### Body mass index

Body mass index was calculated as the ratio of human body weight to height squared in kilograms per square centimeters.

#### HEPA adherence

Two short text messages (short message service (SMS)) were sent once each week to collect data on the number of days during the past week that participants performed circuit training and on how many additional days they performed at least moderate-intensity physical activity for at least 30 min [29].

#### Algometry

Pressure algometry [30] at rest and during standardized static muscle contractions [31] was used to assess PPTs and EIH. The algometer^®^ (Somedics Sales AB, Hörby, Sweden) had a probe area of 1 cm^2^, and the pressure increase was kept at a rate to about 50 kPa/second [30]. The method mainly reflects deep pressure pain and has reasonable reliability [30]. Four physical therapists trained to use the equipment performed assessments.

##### Assessments of PPTs

1. Global pain at rest was rated on visual analogue scale (VAS) (range of 0–100) before PPTs were assessed.
2. PPTs were assessed at rest bilaterally once at six sites (left and right m. supraspinatus, m. gluteus maximus, and the lateral epicondyles). The participants were instructed to indicate when the pressure sensation became painful by pressing the button of the algometer. The mean of the stimulus intensity (in kilopascals) of the six sites was calculated for each participant and reported as the *PPT mean*.
3. Suprathreshold pressure pain sensitivity at rest was assessed at the same six sites. The participants were asked to indicate when the pressure reached the intensity rated as 4/10 (moderate pain) and 7/10 (strong pain) on the Borg category ratio 10 scale (Borg CR 10) [32, 33]. The mean of the stimulus intensity (in kilopascals) of the six sites (4/10 and 7/10) was calculated for each participant and reported as *Suprathreshold 4/10* and *Suprathreshold 7/10.*

##### EIH

The maximum voluntary contraction force (MVC) of the right knee extensors was determined as a basis for the assessment of EIH. MVC was tested using a Biodex Multi-Joint System 4 Pro dynamometer (Biodex Medical Systems, Shirley, NY, USA) while the participant was in a sitting position with hip and knee joints flexed to 90 degrees and hands resting in the lap. Three 5-s measurements of MVC were taken with 1-min rests in between. The highest of the three recorded values was set at each participant’s MVC.

In order to establish baseline values, PPTs at rest were assessed at the right *M. quadriceps* and the left *M. deltoideus* before the contraction.

EIH assessment was based on one submaximal (30% of MVC) isometric contraction of the right knee extensors. *Segmental* EIH was assessed at the contracting *M. quadriceps* and *plurisegmental* EIH at the resting left *M. deltoideus*. The contraction was performed with the participants sitting in the Biodex dynamometer with their hip and knee joints flexed to 90 degrees. They were instructed to perform a right leg isometric knee extension contraction and to maintain it until they were unable to sustain the 30% of their MVC, as indicated by the Biodex dynamometer (maximum 5 min). Throughout the contraction, PPTs were assessed once per site (right *M. quadriceps* and left *M. deltoideus*) every 30 s.

### Data analysis and statistics

Descriptive data are presented as means and standard deviations (SDs) for parametric data and as medians and 25th–75th percentiles for non-parametric data. Differences within groups were calculated with the general linear model (Greenhouse-Geisser for significance) or Friedman test, respectively. The differences at baseline between the total sample and the dropouts (*n* = 40) were calculated with the independent samples *t* test. All analyses were performed using SPSS software (IBM Corporation, Armonk, NY, USA), and the level of significance was set at less than 0.05.

#### EIH

The PPT values from *M. quadriceps* and *M. deltoideus* at baseline, start, middle, and end of the quadriceps contraction were used to calculate EIH. Since baseline PPT values can be expected to vary considerably between individuals [30], the relative changes in PPTs during contraction were analyzed. Thus, as in previous studies [17, 34], the PPTs were normalized by dividing the PPT values of each participant by their first PPT measure at the corresponding site (the first PPT at baseline).

##### Segmental EIH

For normalized PPTs, the values divided by the first PPT at baseline were the second PPT value of the two at baseline (nPPTbase), the first PPT during contraction (nPPTstart), the middle value (if an odd number of PPTs) or the mean value of the two middle values (nPPTmid), and the last PPT (nPPTend) for each individual at *M. quadriceps*. The mean and SD were calculated.

The segmental EIH effects before HEPA, at 1-year follow-up and 2-year follow-up, and the change over time were assessed by analyzing normalized PPTs at the contracting *M. quadriceps* using a repeated measures analyses of variance with the within-factors TIME (four levels: at baseline and three times during contraction: start, middle, and end) and the between-subject factor ASSESSMENT (three levels: before HEPA, at 1-year follow-up, and at 2-year follow-up). Greenhouse-Geisser corrections were used in case of significant test of sphericity.

##### Plurisegmental EIH

The plurisegmental EIH effects were analyzed in the same way as for segmental EIH effects using the normalized PPTs of the distant, resting *M. deltoideus*.

### Ethics approval

All participants were informed about the HEPA intervention and the assessment procedures in writing and consented by coming to the assessments prior to HEPA. The study was approved by the Regional Ethical Review Board in Stockholm (2009/1509–31/1 and 2012/769–32).

## Results

### Participant characteristics

The baseline characteristics of the participants in the total sample and the subsample are described in Table 1. Their health-related quality of life was high, activity limitation was low, and the majority fulfilled criteria for current HEPA at baseline.

### HEPA adherence

At the 1-year follow-up, the participants reported a mean of 58 days (SD 36) of circuit training during the past year and a mean of 252 days (SD 76) of total HEPA, including circuit training. For the second year, a mean of 46 days (SD 40) of circuit training and a mean of 238 days (SD 82) of total HEPA were reported.

### Global pain

The ratings of *global pain* at rest decreased significantly from before intervention to 2-year follow-up (Table 2). Post-hoc analysis showed a decrease from before intervention to 2-year follow-up (*P* = 0.031) and from 1-year to 2-year follow-up (*P* = 0.032).

**Table 2:** Ratings of global pain intensity at rest, individual pressure pain threshold, and suprathreshold pressure pain (4/10 and 7/10) before intervention and at 1-year and 2-year follow-ups

*n* = 30	Before	One-year follow-up	Two-year follow-up	*P* values
Global pain at rest on a scale of 0 to 100, median (25th–75th percentiles)	11 (0–24)	8 (3–25)	6 (0–20)	0.040
PPT mean in kPa, mean (SD)	338 (136)	304 (140)	325 (141)	n.s.
Suprathreshold 4/10 in kPa, mean (SD)	458 (199)	412 (166)	426 (163)	n.s.
Suprathreshold 7/10 in kPa, mean (SD)	647 (302)	539 (230)	560 (193)	0.006

Abbreviations: *n.s.* not significant, *PPT* pressure pain threshold, *SD* standard deviation

### PPT

The *PPT mean* and the *Suprathreshold 4/10* did not change from before intervention to 2-year follow-up, but the *Suprathreshold 7/10* decreased significantly (Table 2), the latter indicating increased pain sensitivity. Post-hoc analysis showed a decrease of the suprathreshold 7/10 from before intervention to 1-year follow-up (*P* = 0.005) and an increase from before intervention to 2-year follow-up (*P* = 0.019).

### EIH

Absolute PPT at rest before the knee extensor contraction (baseline) remained unchanged from the assessment before intervention to the 1-year follow-up and the 2-year follow-up both at *M. quadriceps* (from mean of 510 kPa, SD 254 to mean of 458 kPa, SD 192 to mean of 415 kPa, SD 137) and at *M. deltoideus* (from mean of 235 kPa, SD 113 to mean of 208 kPa, SD 115 to mean of 254 kPa, SD 134).

**Fig. 2:**
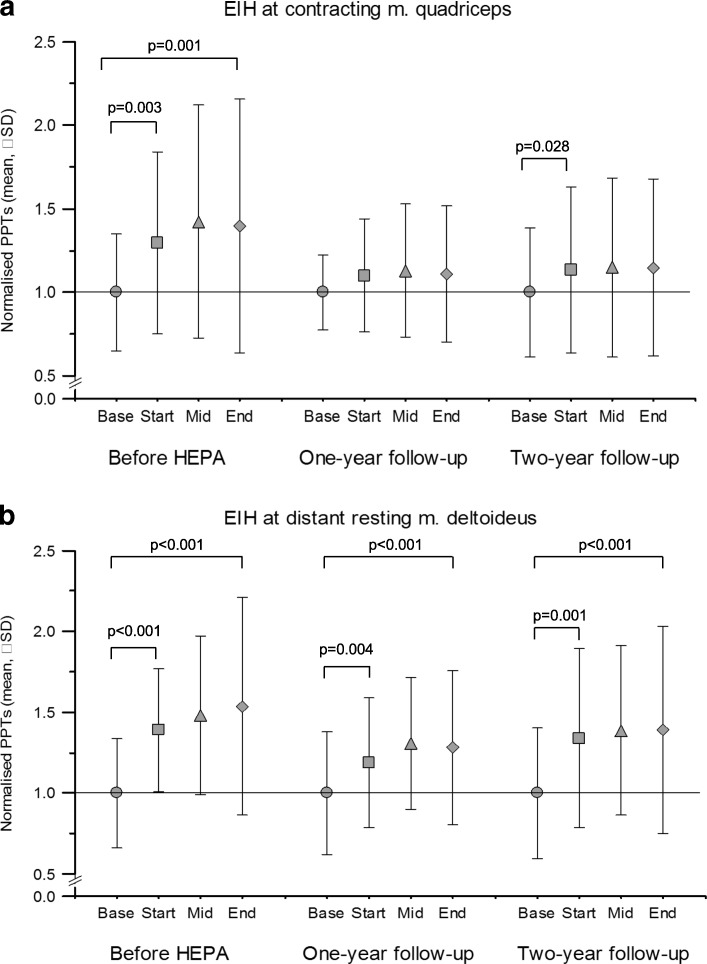
Normalized pressure pain thresholds (PPTs) (means ± standard errors) at the contracting (**a**) *M. quadriceps* (segmental exercise-induced hypoalgesia, or EIH) or the distant resting (**b**) *M. deltoideus* (plurisegmental EIH) at baseline (base), at start, in the middle (mid) and at the end of a standardized *M. quadriceps* contraction, before intervention (health-enhancing physical activity, or HEPA) and at 1-year and 2-year follow-ups among 21 participants. Each PPTvalue was normalized and adjusted (by adding a coefficient) so that the baseline values always corresponded to 1. Values above 1.0 indicate higher PPTs during contraction (that is, EIH activation). Abbreviation: *SD* standard deviation

A functional *segmental EIH* was indicated before intervention by increased PPTs from baseline (mean of 510 kPA, SD 254) to the end of knee extensor contraction (mean of 662 kPA, SD 324) at the contracting *M. quadriceps* (*P* <0.001). This remained unchanged at 1-year and 2-year follow-ups. There was a significant effect for the factor TIME (d = 2.434, f = 11.502, *P* <0.001), but no significant effect was seen for ASSESSMENT, nor was there a significant TIMExASSESSMENT interaction, which indicates a functional EIH at all assessments but no effect of HEPA (Fig. 2a). Post-hoc analysis revealed a significant increase in normalized PPTs from baseline (nPPTbase) to end of contraction (nPPTend) before intervention (1.02 versus 1.42, *P* = 0.001) but not at the 1-year (0.98 versus 1.09, *P* >0.05) or 2-year follow-ups (0.80 versus 0.955, *P* >0.05). Increases in normalized PPTs from baseline (nPPTbase) to the first PPTs during contraction (nPPTstart) were also seen before intervention and at 2-year follow-up but not at 1-year follow-up (Fig. 2a).

A functional *plurisegmental EIH* was indicated before intervention by significantly increased PPTs from baseline (mean of 235 kPA, SD 113) to the end of knee extensor contraction (mean of 357 kPA, SD 174) at the distant resting *M. deltoideus* (*P* <0.001). This remained unchanged at 1-year and 2-year follow-ups. There was a significant effect for the factor TIME (d = 2.334, f = 34.374, *P* <0.001), but no significant effect was seen for ASSESSMENT, nor was there a significant TIMExASSESSMENT interaction, which indicates a functional EIH at all assessments but no effect of HEPA (Fig. 2b). Post-hoc analysis revealed a significant increase in normalized PPTs from baseline (nPPTbase) to end of contraction (nPPTend) alike, both before intervention (0.87 versus 1.41, *P* <0.001) and at 1-year (0.88 versus 1.17, *P* <0.001) and 2-year follow-ups (0.77 versus 1.16, *P* <0.001). Increases in normalized PPTs also occurred before intervention and at 1-year and 2-year follow-ups from baseline (nPPTbase) to the first PPTs during contraction (nPPTstart) (Fig. 2b).

## Discussion

To our knowledge, this is the first study to explore changes in pressure pain sensitivity and activation of pain inhibitory mechanisms among individuals with RA who participated in a long-term HEPA program. The results indicate that 2-year participation in HEPA was associated with clinical improvement, as indicated by a decrease of global pain. However, our hypothesis that HEPA would be associated with reduced pain sensitivity and improved EIH could not be confirmed.

Although we found a statistically significant reduction in pain intensity following HEPA, the magnitude of the pain reduction was small. This would be expected given the participants’ relatively low pain intensity at baseline. However, our participants scored a pain intensity similar to the baseline pain intensities reported in other exercise studies [34] and this most likely reflect that patients volunteering for exercise studies tend to have a relatively low disease impact.

Our findings that two years of HEPA did not influence pain sensitivity (that is, PPT or moderate suprathreshold pressure pain at rest) indicate that long-term HEPA does not reduce pain sensitivity in RA. They also confirm previous results [15–17] suggesting involvement of central pain mechanisms, including central sensitization, in RA pain as indicated by lower PPTs than healthy controls [16, 17]. However, these findings are in contrast to the well-established clinical improvement of pain following exercise [35], which was also confirmed in the present study. Since our results do not favor our hypothesis, pain inhibitory mechanisms other than EIH need to be investigated in order to increase our understanding of the long-term beneficial effects of HEPA on clinical pain among individuals with RA.

There are several possible explanations for the increased sensitivity to strong pressure pain found in the present study. One might be totally unrelated to HEPA but more to previously described continuous sensitization among individuals with RA [15]. It is reasonable to assume that such a process is first indicated at higher levels of suprathreshold pain since its testing includes a component of temporal summation [36]. Thus, it might be argued that the HEPA program favorably influenced sensitivity to pressure pain at lower levels since no increase in pressure pain sensitivity was found for these levels over two years.

In our previous study, using baseline data (before intervention) from the present study [17], we found functioning EIH among both the participants with RA and healthy controls. The present results offer confirmation but also indicate that EIH did not improve significantly during the 2-year HEPA program. One explanation might be that, according to SMS self-reports, the participants did not perform HEPA, particularly circuit training including strengthening exercises, frequently enough. Furthermore, despite clear instructions, they might not have maintained the recommended intensity levels of (the mainly unsupervised) physical activity performed within the study for 2 years. The difficulty to maintain recommended levels of intensity is also supported by our previous study, which found that individuals with RA determine physical activity intensity by standards other than those of health professionals [37]. Studies of aerobic exercise [20, 38] and of resistance exercises [21, 39] indicate that participants with RA seldom maintain high enough levels of physical activity without supervision. This has also been found among individuals with other chronic pain conditions, such as fibromyalgia [40]. The HEPA support program employed in the present study included behavioral change techniques to support not only adoption but maintenance of physical activity [24, 41]. Despite major positive outcome and experiences, the results of the main PARA intervention study clearly illustrate the complexity and difficulty of keeping physical activity at a moderate or higher intensity level over time [29, 42, 43]. A few previous studies on short-term supervised exercise exist but report conflicting results. Two studies including participants with joint pain [19, 34] reported no change in endogenous pain modulation. One of them [34] found no change in pressure pain sensitivity, and the other did not assess pain sensitivity [19]. Another study found a reduction of pressure pain sensitivity after a supervised 12-week exercise program and the authors suggested that this was likely attributed to a high exercise intensity and high adherence to the program [44]. All together, our present findings may indicate that physical activity on a level that is feasible enough for people with RA to maintain over time outside a health-care context does not correspond to that required for improving endogenous pain modulation and reducing pain sensitivity.

The strengths of our study are the long-term perspective and the fact that the HEPA program was performed in a natural setting with the participants paying their own expenses, thus resembling reality to a great extent. There was a fairly high dropout rate as the result of poor adherence, and there were incomplete data due to an administrative error. Furthermore, since the dropout analysis indicated no major differences between those meeting the inclusion criteria for the present study and those with incomplete data or not meeting the criteria for HEPA for two years (or both), there should be no major influence on external validity. Long-term exercise interventions using multiple assessment methods require comprehensive administrative and methodological procedures, resulting in limited numbers of participants. Thus, although a total sample of 30 participants with RA might be considered a limitation to our study, it resembles the size of samples in previous studies investigating endogenous pain modulation in response to exercise in rheumatic diseases [19, 34]. The choice of methodology for the present study might have influenced our results. Assessing individual pain-related central mechanisms is complex because several methods measure different aspects. Thus, the long-term effect of HEPA on pain modulation was assessed by segmental and plurisegmental EIH using normalized PPTs during static muscle contraction [30]. An alternative method would have been conditioned pain modulation, which measures the pain-inhibits-pain mechanism [19] used to assess endogenous pain modulation. However, although test results obtained with the two methods do not correlate [45], results from studies using either of the two methods in participants with RA seem to point in the same direction (that is, normally functioning conditioned pain modulation [15] and EIH [17]).

The present study explored the influence of long-term HEPA on pain sensitivity and EIH. Future studies should investigate how long-term physical activity with different intensities contributes to changes in EIH among individuals with RA. Furthermore, studies are needed to explore the role of long-term sensitization and the natural long-term course of EIH development among individuals with RA.

## Conclusions

The present results indicate that participation in a long-term HEPA program did not reduce pain sensitivity at rest or improve EIH among participants with RA but was associated with reduction of global pain ratings. Thus, our results do not favor the hypothesis that long-term HEPA reduces pain sensitivity by improving descending pain inhibition among individuals with RA.

## Abbreviations

EIH: Exercise-induced hypoalgesia
HAQ-DI: Stanford Health Assessment Questionnaire Disability Index
HEPA: Health-enhancing physical activity
IPAQ: International Physical Activity Questionnaire
MVC: Maximal voluntary contraction
nPPTbase: Normalized value of the second pressure pain threshold at baseline
nPPTend: Normalized value of the last pressure pain threshold during contraction
nPPTmid: Normalized middle value of the pressure pain thresholds during contraction
nPPTstart: Normalized pressure pain threshold at the start (first 5 s) of contraction
PARA: Physical activity in rheumatoid arthritis 2010 study
PPT: Pressure pain threshold
RA: Rheumatoid arthritis
SD: Standard deviation
SMS: Short message service
